# Novel Air Flow Meter for an Automobile Engine Using a Si Sensor with Porous Si Thermal Isolation

**DOI:** 10.3390/s121114838

**Published:** 2012-11-02

**Authors:** Emmanouel Hourdakis, Panagiotis Sarafis, Androula G. Nassiopoulou

**Affiliations:** Institute of Microelectronics, NCSR Demokritos, Terma Patriarchou Grigoriou, 15310 Aghia Paraskevi, Greece; E-Mails: mhour@imel.demokritos.gr (E.H.); pansarafis@imel.demokritos.gr (P.S.)

**Keywords:** air flow meter, thermal mass flow sensor, porous Si thermal isolation, automobile engine

## Abstract

An air flow meter for measuring the intake air of an automobile engine is presented. It is based on a miniaturized silicon thermal mass flow sensor using a thick porous Si (Po-Si) layer for local thermal isolation from the Si substrate, on which the sensor active elements are integrated. The sensor is mounted on one side of a printed circuit board (PCB), on the other side of which the readout and control electronics of the meter are mounted. The PCB is fixed on a housing containing a semi-cylindrical flow tube, in the middle of which the sensor is situated. An important advantage of the present air flow meter is that it detects with equal sensitivity both forward and reverse flows. Two prototypes were fabricated, a laboratory prototype for flow calibration using mass flow controllers and a final demonstrator with the housing mounted in an automobile engine inlet tube. The final demonstrator was tested in real life conditions in the engine inlet tube of a truck. It shows an almost linear response in a large flow range between –6,500 kg/h and +6,500 kg/h, which is an order of magnitude larger than the ones usually encountered in an automobile engine.

## 1. Introduction

In recent years sensors have become a necessary component of automotive electronic systems. The number of sensors utilized by the automotive industry is fast increasing in order to meet requirements such as correct engine control for optimum fuel consumption, control of emissions regulations [1–3], *etc*. More specifically, air flow meters measure the air entering a fuel-injected internal combustion engine. This is necessary information for the engine control unit to balance and deliver the correct fuel mass to the engine, thus regulating the air-fuel mixture for achieving fuel economy, enhanced engine performance and reduction of emissions [1,2].

There are several different types of sensors used in air flow meters. One category of sensors uses an indirect measurement such as the speed of the engine and the engine temperature and pressure to determine the air flow [4]. These are not direct measurements and are therefore susceptible to errors both by the multiple measurements required and by the assumptions made during the determination of the air flow. Other methods, such as the hot-wire method, use a direct measurement of the air flow based on thermal heat loss principles [5]. These types of methods, though, cannot account for changes in the direction of the flow, so they cannot be considered as true mass air flow measurements. Changes in the direction of the air flow are common during low engine speed operation. Existing true mass air flow meters use sensors that are based on micromechanically fabricated free standing membranes [6,7]. These meters are capable of detecting flow direction reversal. In addition, the free standing membranes assure very effective local thermal isolation from the Si substrate [8–10], thus reducing power consumption on the heater. Their disadvantage is that the free standing membranes are relatively fragile and not compatible with further Si processing.

In this work we present an air flow meter using a silicon thermal flow sensor based on a robust, planar, and very effective local thermal isolation strategy based on porous Si local thermal isolation [11,12]. Highly porous Si is a nanostructured material presenting many interesting properties [13,14], including a thermal conductivity that is more than two orders of magnitude lower than that of bulk crystalline Si [15]. It has been successfully used by different groups in a variety of sensors and other applications [16–19]. The sensor used in this work has a thick porous Si layer locally formed on the Si substrate, on which the sensor active elements are integrated. It has been developed within the author's group [11,12] and has been described in detail elsewhere [15,20–24]. It is versatile and can be used in several applications by adapting its packaging and housing to the corresponding application. It has been already used as the thermal sensor of a respiration control flow meter [21], as an acceleration sensor [22] and as a low power consumption gas sensor [23] for use in an explosive environment. The sensor is very sensitive to air flow, it is low cost and all its fabrication steps, apart from porous Si formation, use batch Si processing technology. It is very small and fast responding. The porous Si layer is locally formed on the Si wafer on pre-selected areas at the beginning of the process and the wafer is then subjected to standard Si processing steps. The porous Si surface area is planar with the rest of the wafer and it assures local thermal isolation from the Si substrate that can be as effective as a free standing membrane if the porous Si layer is sufficiently thick.

In the present application the above Si thermal flow sensor is used as the sensing element of an air flow meter for measuring the mass flow rate of air entering a fuel-injected internal combustion automobile engine. It will be shown that the response of the flow meter is almost linear with flow (to within 18% from the ideal linear curve) in the entire flow range of interest for this application. As demonstrated previously, the sensor is fast responding, showing a response time less than ∼1 ms [24].

## 2. Flow Sensor Design and Fabrication

The flow sensor used in the developed flow meter is a miniaturized Si sensor (Si die dimensions: 1 mm × 1.4 mm) composed of the following parts:

- A thick porous Si layer of lateral dimensions 300 μm × 600 μm, on which the sensor active elements are integrated
- A boron-doped polycrystalline silicon resistor used as heater and lying in the middle of the porous Si surface area
- Two series of thermocouples (thermopiles) on each side of the heater. The thermocouples are made of p-type polycrystalline Si in contact with Al. The “hot” contact of the thermocouples lies on the porous Si layer, while their “cold” contact lies on the bulk crystalline Si substrate outside the porous Si area.

**Figure 1.**
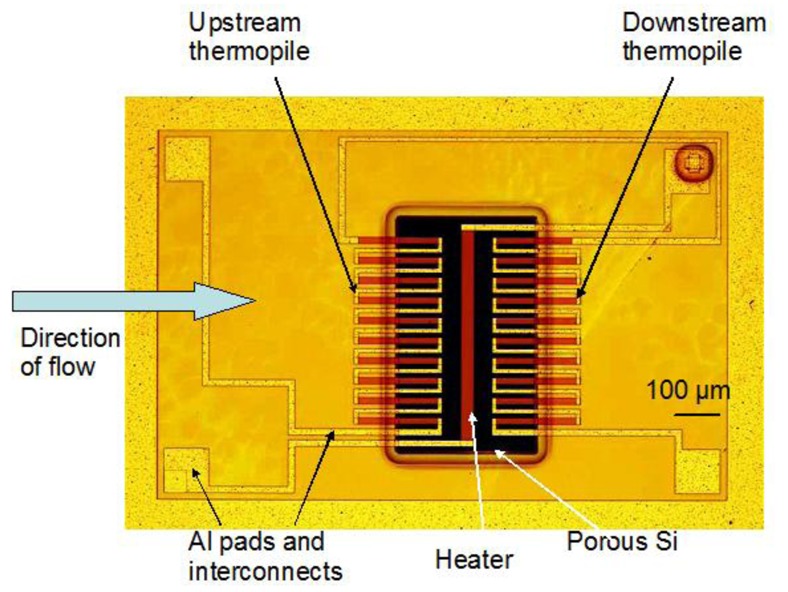
Schematic of the sensor surface area depicting the porous Si area, the heater and two series of thermocouples (upstream and downstream thermopile) and contact pads and interconnects. The flow direction is perpendicular to the heater and is indicated by the horizontal arrow.

An optical micrograph of the sensor surface area is presented in Figure 1. The porous Si layer is illustrated as the black rectangular area in the schematic. The heater and two series of thermocouples (upstream thermopile and downstream thermopile) are also indicated. The air flows above the heater in a flow channel, the direction of flow being perpendicular to the heater, as indicated by the horizontal arrow in the schematic.

**Figure 2.**
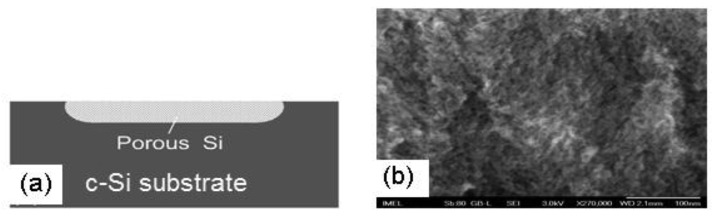
(**a**) Cross sectional schematic representation of the porous Si layer on the Si substrate. (**b**) Cross sectional scanning electron micrograph of the porous Si layer depicting its sponge-like structure.

The porous Si layer is fabricated locally on the Si substrate by electrochemical dissolution of bulk crystalline Si in an aqueous HF solution. P-type Si with resistivity in the range of 6–8 Ω.cm is used. More details on porous Si formation can be found elsewhere [11–24]. The porosity and structure of the material depend strongly on the electrochemical conditions used for its fabrication. A schematic of the cross section of the porous Si layer on the Si substrate is depicted in Figure 2(a), while Figure 2(b) shows a cross-sectional scanning electron microscopy (SEM) image of the porous Si layer, depicting its sponge-like porous structure.

The porosity of the material used was ∼70% and its thermal conductivity of the order of ∼1.2 W/mK [15,24]. Due to its low thermal conductivity, the porous Si layer assures that the temperature above the heater and the hot contact of the thermocouples changes rapidly with the temperature of the air fluid above the sensor, induced by the gas flow. Thus, under flow, the temperature of the “hot” contact of the thermocouples changes with flow, while that of the “cold” contact stays almost stable due to the large thermal conductivity of Si (145 W/mK). A temperature difference is thus created between “hot” and “cold” contacts of the thermocouples, resulting in a voltage drop across the thermopiles, proportional to the temperature difference of the gas above the sensor. The flow direction above the sensor active elements is perpendicular to the long axis of the heater, as illustrated in Figure 1. One series of thermocouples is in the flow “upstream”, while the second one in the flow “downstream”. Consequently, under flow the “upstream” thermopile is cooled down by the fluid, while the “downstream” thermopile is heated up, due to displacement by the flow of the hot air above the heater. The voltage difference created at the output of the two thermopiles is of opposite sign [20]. Using a differential connection of the two thermopile output signals, the total output signal is the sum of the absolute value of the two thermopile signals, thus increasing the sensor sensitivity. The sensors are fabricated on 4 inch Si wafers, each containing 5.000 sensor dies.

## 3. Sensor Characterization under Static Conditions (No Flow)

**Figure 3.**
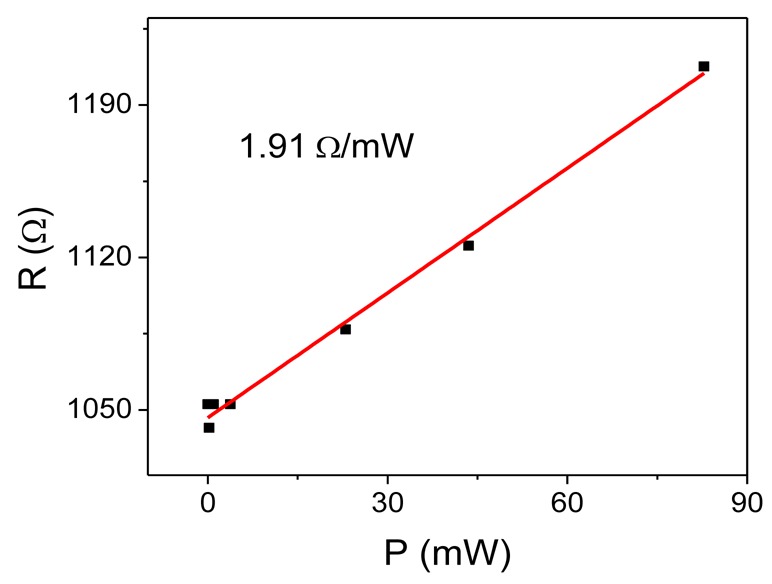
Typical resistance versus power characteristic of the polysilicon heater on porous Si. The red line is a linear fit to the experimental data. The slope of the curve (resistance variation with power) is found to be 1.91 Ω/mW.

A typical characterization of the sensor under static conditions (without flow) consists of measuring the heater resistance variation as a function of the applied electrical power to it (R-P characteristic). A typical R-P characteristic is shown in Figure 3. The relation between resistance and applied power is linear and it reflects the change of temperature of the heater with the applied power. The slope of this characteristic is much larger (by at least one order of range) compared to the slope of a similar curve of the same heater on bulk crystalline Si [20]. This is due to the effective thermal isolation of the porous Si layer. The resistance change with power of the 1,050 Ω heater of Figure 3 is 1.91 Ω/mW, while that of the same resistor on bulk crystalline Si was found to be 0.16 Ω/mW [24].

## 4. Air Flow Meter Design and Fabrication

**Figure 4.**
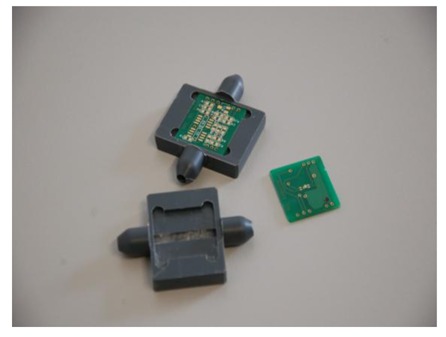
Picture of the main parts of the laboratory prototype of the air flow meter, comprising the housing with its inlet, outlet and central semi-circular flow tube and the PCB with its one side dedicated to the sensor mounting and the other side to the mounting of the electronic components.

**Figure 5.**
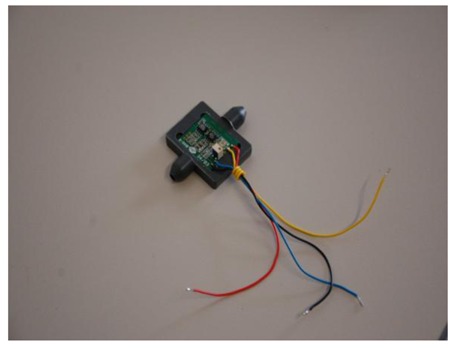
Picture of the full meter comprising the housing and the PCB with the electronics and the sensor on it mounted on the housing.

The flow meter was designed for use in the air inlet tube of a car engine. It consists of the following parts:

- A two-sided printed circuit board (PCB) with the sensor mounted on one side of it and the readout and control electronics on the other. The electronics were designed to control the application of a constant power on the heater and to amplify the differential signal output from the two thermopiles. The amplification factor was variable and adjustable depending on the flow range required in each application. The sensor die was mounted on the PCB and electrical connection between the contact pads of the die and the PCB was made by Al wirebonds. A molding technique by Boschman Technologies was applied to encapsulate the wirebonds in order to protect them from damage caused by large air flows or mechanical vibrations, while leaving the active area of the sensor die open.
- A first laboratory prototype was designed, in order to fully characterize the meter under laboratory conditions, using mass flow controllers for flow calibration. The housing was composed of an inlet and an outlet tube with internal diameter ∼4 mm and a central part on which the PCB comprising the sensor was mounted. This central part comprised a semi-circular tube with the same diameter as the inlet and outlet tubes (∼4 mm), and served as bypass to the air flow. The central part of the housing was designed to accommodate the PCB (lateral dimensions 2 cm × 2 cm), with the sensor on the PCB situated in the middle of the semi-circular flow tube and its active area exposed to the flow. The design of the electronics has taken into account the need for size minimization of the PCB. A picture of the housing, the PCB without the electronics and the PCB mounted on the housing, is shown in Figure 4. Figure 5 shows the full meter with the housing and the PCB with the corresponding electronics components mounted on it.
- A final demonstration unit was designed and fabricated, comprising a second housing with the same flow channel diameter, but with a different external design. This housing was adapted to the flow tube of the air inlet of an automobile engine, together with the automobile flow tube. The final demonstrator was directly tested on a truck engine under real life conditions, in comparison with a commercial air flow meter. The corresponding results will be presented below.

## 5. Air Flow Meter Design and Fabrication

### 5.1. Calibration of the Laboratory Prototype under Nitrogen Flow

**Figure 6.**
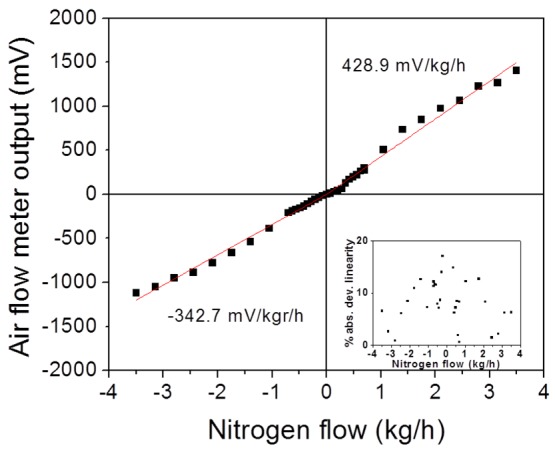
Calibration curve of the air flow meter under nitrogen flow in forward and reverse flow directions, depicting quite symmetrical behavior in both directions. The points are the experimental results, while the red lines represent the linear fit to these points. The air flow meter response is remarkably linear with flow. The inset shows the absolute percent deviation from linearity for all data points. The deviation is less than 18% for all data points.

Using the above described laboratory prototype flow meter and calibrated mass flow controllers, a calibration curve of the output of the air flow meter (in units of mV) as a function of the flow through the flow channel (Q_FlowChannel_ in units of standard liters per minute (slpm)) was recorded. The slpm units were then converted into kg/h that are usually used in automotive air flow meters. The electronics of the flow meter were biased at 9 V. An example of calibration curve is shown in Figure 6.

It can be easily seen from Figure 6 that the flow meter response is linear with flow in both the forward and reverse directions. The difference between the slopes of the forward and reverse flows comes from an asymmetry of the positioning of the sensor die on the PCB (the sensor die is closer to one edge of the PCB than the other). In the inset of Figure 6 the absolute deviation from linearity in % is presented for all data points. It can be clearly seen that all data points deviate from linearity by less than 18%. For calculating the flow conditions under which the flow meter is operated, we consider the Reynolds number (Re) for the flow channel of the housing used, given by [25]:

(1) $$\text{Re}=\frac{Q_{\text{FlowChannel}}D_{\text{eff}}}{v_{\text{air}}A}$$

where Q_FlowChannel_ is the flow, v_air_ = 15.68 × 10^−6^ m^2^/s is the kinematic viscosity of air at 300 K, A is the cross sectional area of the flow channel and D_eff_ is the hydraulic diameter of the flow channel, given by:

(2) $$D_{\text{eff}}=\frac{4A}{P}$$

where P is the wetted perimeter of the flow channel cross section. We note that, as was mentioned before, the shape of the flow channel cross section is a half-circle of diameter D = 4 mm. Combining the above we have:

(3) $$\text{Re}=\frac{8Q_{\text{FlowChannel}}}{3\pi Dv_{\text{air}}}$$

The condition for a laminar flow is given by: Re < 2,300 which translates to Q_FlowChannel_ < 10.1 slpm. For fully turbulent flow the condition is: Re > 10,000 or Q > 44 slpm. From the above it results that the fabricated flow meter with its 4 mm diameter flow channel operates under laminar flow for flows below ∼10 slpm (∼0.7 kg/h), while for flows between 10 and 44 slpm (0.7 and 3.07 kg/h) it operates under mildly turbulent flow conditions.

### 5.2. Testing of the Air Flow Meter Mounted in the Air Inlet Tube of a Truck

**Figure 7.**
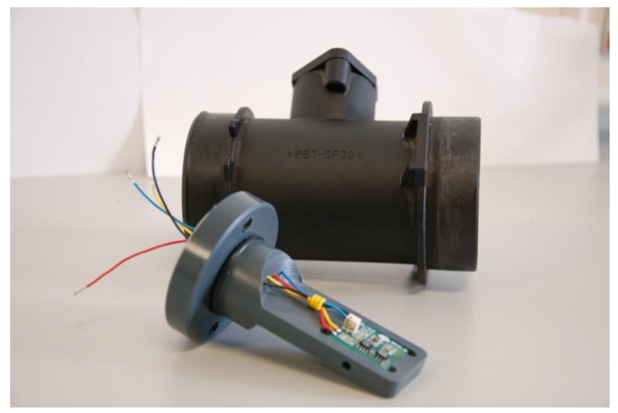
Picture of the final demonstrator of the air flow meter and the automobile engine inlet tube (black tube) in which it is mounted.

**Figure 8.**
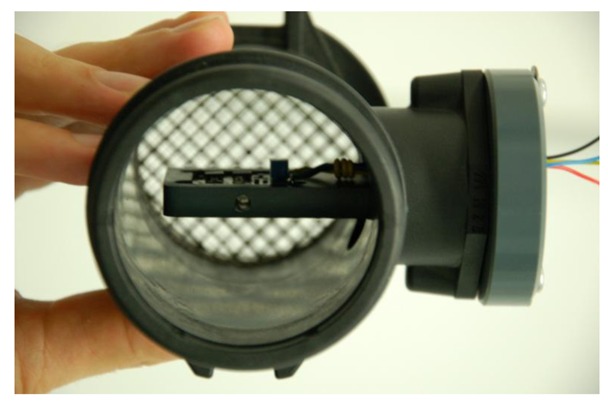
View of the interior of the automobile engine inlet tube with the air flow meter mounted in it.

The final demonstrator with the air flow meter was finally mounted in a commercial automobile engine inlet tube for real life measurements. A picture of the complete air flow meter with the second housing and the automobile engine inlet tube in which it is mounted for real life measurements is presented in Figure 7. Figure 8 shows a cross sectional view of the engine inlet tube with the air flow meter in it.

**Figure 9.**
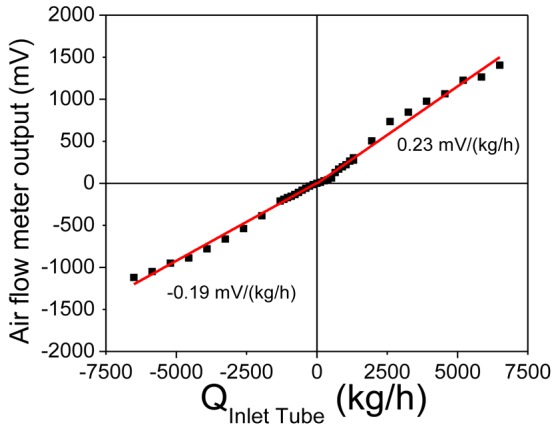
Calibration curve of the air flow meter output as a function of air flow through the engine air inlet tube. Black squares are the experimental data, while the red lines represent the linear fit to these data in the forward and reverse flow directions.

The fact that the flow channel dimensions of the second housing designed for the automobile engine are identical to those of the laboratory prototype allows for an estimated extrapolation of the results of Figure 6 in the case of air flow through the engine inlet flow tube. The amount of air flowing in the flow channel of the sensor housing is a given percentage of the air flowing in the engine inlet tube. More specifically:

(4) $$Q_{\text{InletTube}}=Q_{\text{FlowChannel}}\frac{A_{\text{InletTube}}}{A_{\text{FlowChannel}}}\frac{\rho_{\text{air}}}{\rho_{\text{nitrogen}}}$$

where Q_FlowChannel_ is the flow through the housing flow channel, A_InletTube_ is the cross sectional area of the engine inlet tube and A_FlowChannel_ is the cross sectional area of the flow channel, ρ_air_ = 1.205 kg/m^3^ and ρ_nitrogen_= 1.165 kg/m^3^ are the densities of air and nitrogen at 300 K respectively. The diameter of the inlet tube is 120 mm, while the diameter of the semi-circular tube of the air meter flow channel is 4 mm so that the multiplication factor between the air flow in the air flow meter and that of the engine inlet tube is equal to 1,861.8. Using the above multiplication factor, the estimated calibration curve of the air flow in the air inlet tube of the automobile engine is illustrated in Figure 9.

From Figure 9 it is clear that the air flow meter calibrated for the air flow in the large engine air inlet tube shows the same linear response as the laboratory prototype. This range is one order of magnitude larger than the flow range typically encountered in an automobile engine with an inlet tube of 120 mm diameter. The presented calibration curve of Figure 9 is just an estimate of the calibration curve under real-life conditions. In fact, the flow range estimated is much larger than the flow ranges that can be experimentally achieved in the laboratory. However, this curve was experimentally confirmed by the measurements performed in the inlet of a truck engine in comparison with a commercial system for the range of interest in the automotive industry, a range much smaller than the estimate presented in Figure 9. These results are presented below.

The output signal of the air flow meter can be easily increased by increasing the amplification provided by the electronic circuit, thus increasing the slope of the corresponding calibration curve. The fact that the system response with flow is linear allows for the direct increase of the output signal by increasing the amplification. An amplification factor of 100 was used in Figures 6 and 9, resulting in a slope of 0.23 mV/(kg/h) in the forward flow direction and –0.19 mV/(kg/h) in the reverse flow direction.

**Figure 10.**
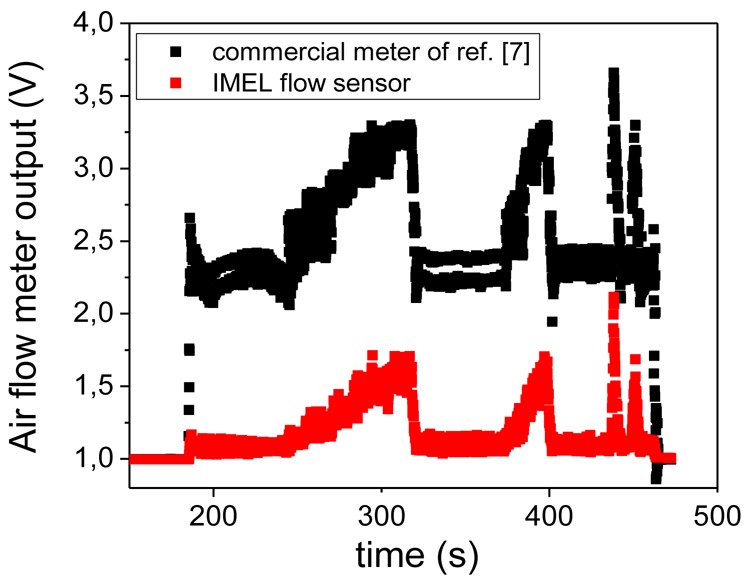
Air flow meter output as a function of time for the above presented air flow meter (red open squares) and the commercial meter of [7] used (black squares). The response of the two systems was monitored by arbitrarily pushing the gas pedal.

Finally, the above air flow meter was tested under real life conditions by mounting it in the inlet of a truck engine in series with a commercial flow meter. The comparison was made with the commercial system of [7]. The signal from both meters was recorded as a function of time as the engine was randomly revved up and down. The results of this test for both our flow meter and the commercial meter appear in Figure 10. For a direct comparison of the two meters, the signal from our air flow meter was normalized to the one of the commercial meter by multiplying by a factor of 5.9. This scaling was performed so that the value of the output for both meters was equal up to the time where the engine started to be revved up and down. From Figure 10 we deduce that our air flow meter (appearing as present air flow meter in Figure 10) follows the same signal variations as the commercial one.

**Figure 11.**
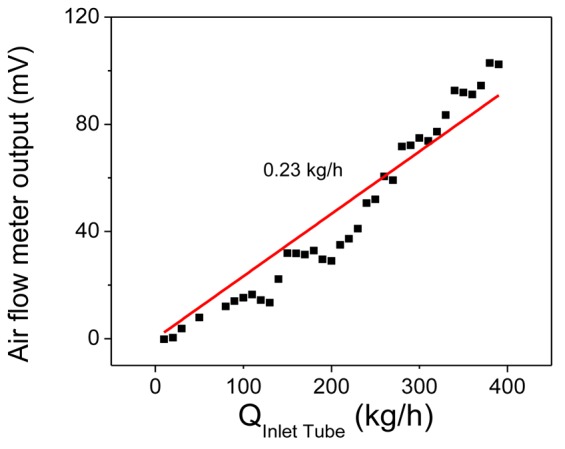
Calibration curve of the present air flow meter mounted in the inlet tube of a truck engine as a function of the measured flow using the commercial air flow meter of [7], mounted in the same truck engine tube. The points are the experimental results, while the red line is the linear fit to these data.

Using the first part of the measurements of Figure 10 (for the first 300 s) and the calibration curve of the commercial flow meter [7], a calibration curve for our above described air flow meter has been constructed and is presented in Figure 11. The noise from the commercial meter has been averaged out. The calibration curve of Figure 9 appears to deviate slightly from linearity. This is attributed to the error introduced by the non-linearity of the commercial air flow meter [7] used for reference. We used a linear fit of the data to compare the results of Figure 11 with those of Figure 9. This linear fit, represented by the red line in Figure 11, reveals a slope for this curve of 0.23 kg/h. This slope, calculated from data collected under real life conditions on a truck under air flow, is identical to the slope obtained for the laboratory prototype (also 0.23 kg/h, calculated from Figure 9), calibrated using mass flow controllers and nitrogen flow for a flow range more than one order of magnitude larger than the real life conditions of the track. This is very strong evidence that our flow meter behaves as anticipated under real life conditions and for a flow range more than one order of magnitude larger than the range tested with the laboratory prototype. This is a proof that the extrapolation of the measurement in the air meter flow channel (bypass tube) to the large air inlet tube of the automobile is a viable method for obtaining the correct calibration curve in this case.

## 6. Conclusions

We have reported a novel true mass air flow meter for measuring the mass air flow rate entering an automobile engine and compared its response to an existing commercial air flow meter. This air flow meter is based on a silicon thermal air mass flow sensor using local thermal isolation by a thick porous Si layer. This technology results in a miniaturized, robust, sensitive, and low cost Si sensor. The developed air flow meter shows a linear response in a very wide range of flows between –6,500 kg/h to +6,500 kg/h and it maintains its linearity for flows ranging from laminar to the onset of fully turbulent flow. It also distinguishes between forward and reverse flows.

## References

[1] Fleming W.J. (2001). Overview of automotive sensors. IEEE Sens. J..

[2] Fleming W.J. (2008). New automotive sensors—A review. IEEE Sens J..

[3] Docquier N., Candel S. (2002). Combustion control and sensors: A review. Prog. Energ. Combust..

[4] Delphi Corp. Delphi Manifold Absolute Pressure/Manifold Air Temperature (MAP/MAT) Sensors. http://delphi.com/shared/pdf/ppd/sensors/map-mat-sensors.pdf.

[5] Brasseur G. Robust Automotive Sensors.

[6] Lembke M., Hecht M., Konozelmann U. (2004). Method and device for determining gas flow.

[7] Hot-Film Air-Mass Meter HFM 5. http://apps.bosch.com.au/motorsport/downloads/sensors_airmass.pdf.

[8] Cubucku A.S., Zemickela E., Buerklin U., Urban G.A. (2010). A 2D thermal flow sensor with sub-mW power consumption. Sens. Actuators A: Phys..

[9] Xue N., Yan W.P. (2012). A silicon-glass-based microfabricated wide range thermal distribution gas flow meter. Sens. Actuators A: Phys..

[10] Hendrich F., Kliche K., Storz M., Billat S., Ashauer M., Zngerle R. (2010). Thermal flow sensors for MEMS spirometric devices. Sens. Actuators A: Phys..

[11] Nassiopoulou A.G., Kaltsas G. (1998). Integrated gas flow sensor based on porous Si micromachining.

[12] Nassiopoulou A.G., Pagonis D., Kaltsas G. (2007). Low power silicon thermal sensors and microfluidic devices based on the use of porous sealed air cavity technology or microchannel technology.

[13] Canham L. (1997). Properties of Porous Silicon.

[14] Sailor M.J. (2012). Porous Si in Practice: Preparation, Characterization, Properties.

[15] Nassiopoulou A.G., Kaltsas G. (2000). Porous silicon as an effective material for thermal isolation on bulk crystalline silicon. Phys. Stat. Sol. A.

[16] Mathew A., Pandian G., Bhattacharia E., Chadha A. (2009). Novel applications of Si and porous Si based EISCAP biosensors. Phys. Stat. Sol. A.

[17] Winans J.D., Lee J.Y., Veeramachaneni B., Hu S., Kawamura D., Witt K., Hirschman K.D., Fauchet P.M. (2011). Isolated silicon waveguides via porous silicon formation by targeted fluorine doping. Phys. Stat. Sol. A.

[18] Desplobain S., Gautier G., Ventura L., Bouillon P. (2009). Macroporous silicon hydrogen diffusion layers for micro-fuel cells. Phys. Stat. Sol. A.

[19] Granitzer P., Rumpf K., Poelt P., Krenn H. (2009). Porous Si/metal nanocomposite with tailored magnetic properties. Phys. Stat. Sol. A.

[20] Kaltsas G., Nassiopoulos A.A., Nassiopoulou A.G. (2002). Characterization of a silicon thermal gas-flow sensor with porous silicon thermal isolation. IEEE Sens. J..

[21] Kaltsas G., Nassiopoulou A.G. (2004). Gas flow meter for application in medical equipment for respiratory control: study of the housing. Sens. Actuators A: Phys..

[22] Goustourides D., Kaltsas G., Nassiopoulou A.G. (2007). A silicon accelerometer without solid proof mass using porous Si thermal isolation. IEEE Sens. J..

[23] Tsamis A., Nassiopoulou A.G., Tserepi A. (2003). Thermal properties of suspended porous Si micro-hotplates for sensor applications. Sens. Actuators B: Chem..

[24] Kaltsas G., Nassiopoulou A.G. (1999). Novel C-MOS compatible monolithic silicon gas flow sensor with porous silicon thermal isolation. Sens. Actuators A: Phys..

[25] Incropera F.P., DeWitt D.P., Bergman T.L., Lavine A.S. (2006). Thermal Analysis and Convention Correlations. Fundamentals of Heat and Mass Transfer.

